# The Production of the PHAR-QA Competence Framework †

**DOI:** 10.3390/pharmacy5020019

**Published:** 2017-04-01

**Authors:** Jeffrey Atkinson

**Affiliations:** 1 Lorraine University, 5 rue Albert Lebrun, 54000 Nancy, France; jeffrey.atkinson@univ-lorraine.fr; Tel./Fax: +33-383-273-703; 2 Pharmacolor Consultants Nancy, 12 rue de Versigny, 54600 Villers, France

**Keywords:** pharmacy, education, competences, framework, methodology

## Abstract

This article describes the background and methodology of the PHAR-QA (Quality Assurance in European Pharmacy Education and Training) project that produced a competence framework for pharmacy education and practice in the EU. In order to produce a harmonized competence framework that could be accepted within the EU situation, we developed a two-stage Delphi process centred on two expert panels. A small panel of academics produced the competence framework that was then validated by the rankings of a large panel consisting of representatives of the EU pharmacy community. The main aspects of this process are developed in this article.

## 1. Introduction

This article describes the background and methodology of the PHAR-QA (Quality Assurance in European Pharmacy Education and Training) [1] project and provides several ideas on the methodology for those wishing to undertake a similar exercise. The results of the PHAR-QA have been published [2] and the reader of this article should refer to that paper for all details of the methodology, results, conclusions and perspectives.

PHAR-QA was planned to produce a competence framework for pharmacy education and training (PET) in Europe. It was a follow-up to the PHARMINE (Pharmacy Education in Europe) project [3] that surveyed the present situation of education and training in European pharmacy departments, both in terms of “structure” (resources and management, staff and student numbers, timing, duration of courses, subject areas taught, etc.), and “competences” (knowledge and ability to perform as pharmacy practitioners, quality assurance, etc.). Both projects took into account the wide diversity of pharmacy practice (community, hospital, industry, administrative, etc.) in the EU.

## 2. Background

### 2.1. Rationale: Why Carry Out the PHAR-QA Project

A first reason for considering the PHAR-QA project was the observation that European PET is extremely varied as far as structural aspects are concerned, and, furthermore, very little of it is based on competence learning [4]. The situation has not changed since the survey carried out by Pierre Bourlioux and the European Association of Faculties of Pharmacy, in the EU in 1994 [5].

The above situation is paradoxical in that there exists a European directive on the harmonization of the sectoral profession of pharmacy with recommendations for PET [6]. However, this—as all directives—is the result of the EU comitology process which tends to be aimed more towards resources and management rather than to ability. Thus, regarding PET, the EU directive focuses on 10 activities (e.g., “preparation of the pharmaceutical form”) and 14 course subjects (e.g., “plant and animal biology”), with reference to wide competences (e.g., “adequate knowledge of medicines”).

The above situation is unfortunate as one of the fundamental laws of the EU is the right of patients in the EU to efficient healthcare, regardless of the member state in which it is proffered. This is embedded in the EU directive on patients' rights to cross-border healthcare [7].

A second observation was that there is no harmonized European system for implementation and evaluation of competence-based learning and training in pharmacy [8]. In a survey on existing quality assurance and accreditation systems in 10 EU member states, we found that the existing schemes are based mainly on management and resources and little on competences. Furthermore, existing schemes are national and obligatory. Thus, in the EU, PET, as education and training in other sectors of healthcare, is organized on a confederal rather than a federal basis. A federal system assigns more power to the central government, whereas a confederate system reserves most of the power for the member states. This allows, therefore, substantial independence on the part of the member states regarding the way in which they organize PET in their specific country. Any attempt to impose a rigid, obligatory system for PET would probably fail given this European situation. This is why PHAR-QA proposed a harmonized, consultative system based not on management and resources but on competences. The ways in which pharmacy practice competences are gained will vary from one member state to another.

### 2.2. The Starting Points: Existing Competence Frameworks

In order to avoid the “NIH” (“not invented here”) syndrome, a review of existing competence frameworks for PET, and those for education and training in other healthcare areas (medicine, dentistry, etc.), was carried out by A. Sanchez-Pozo and D. Rekkas (see chapter by A. Sanchez-Pozo in this special book edition). We also considered the recommendations outlined in the EU directive on the sectoral profession of pharmacy [6]. On the basis of the review, a list of proposed competences for pharmacy practice was produced.

## 3. Methodology

### 3.1. Type of Competence

Proposed competences were of two types: “knowledge/being aware of” and “ability/capable of doing”. The first type of competence corresponds to the two lower levels (“knows/knowledge” and “knows how/competence”) of Miller’s triangle [9], the second to the two upper levels (“shows how/performance” and “does/action”). These two types of competences were proposed (“knowledge” and “ability”) as the consortium considered that in some areas of pharmacy practice all students should be “aware of” without necessarily being “capable of doing”. One example is “knowledge of design, synthesis, isolation, characterisation and biological evaluation of active substances”. The consortium considered that students should be aware of such aspects of industrial pharmacy and R&D, without necessarily being capable of applying the methodology to synthesise, evaluate, etc., themselves. Competences were ranked on a 4-point ranking scale: from “not important/can be ignored” to “essential/obligatory”, proposed by the MEDINE (*Medical education in Europe)* consortium [10] with whom PHAR-QA collaborated.

### 3.2. The Two-Panel Delphi Process: The Small and Large Panels

The process used in the PHAR-QA project was a modified Delphi, two-stage process involving two panels: firstly, a small panel consisting of the 13 consortial members whose names and affiliations are given at the end of this article. All were academics with substantial experience in PET. The initial function of the small panel was to produce a questionnaire on the basis of the report on starting points (see Section 3.1. above). This was produced by three Delphi rounds. The second function of the small panel was to evaluate the results of the first round of the large panel Delphi (see below), and on the basis of this, to produce a second refined version for examination by the large panel. The large panel consisted of pharmacy students, academic staff and professionals (community, hospital, industrial pharmacists and pharmacists working in other fields). The large panel had two main functions: firstly, to rank the competences in two anonymous, Delphi rounds; secondly, to ensure the validation by the global pharmacy community of a competence framework produced by academics.

This large panel paradigm has been used but rarely in the production of competence frameworks in healthcare sciences; notable exceptions being the PHAR-QA project and MEDINE. As in MEDINE, it was used here in order to facilitate the acceptance of the final competence framework by both the professional community and university circles. This is a cardinal point in the PHAR-QA study. The major difference with MEDINE was that PHAR-QA ran two rounds of large panel ranking whereas MEDINE ran only one. This posed the question of the repeatability of the results using the PHAR-QA methodology (see Section 3.4 below).

### 3.3. Iteration versus Anonymity—Implications for the Repeatability of the Results

In order to ensure the anonymity of the respondents of the large panel, the option of collecting individual emails in the first round then using them in the second was not taken. The second round questionnaire was sent to the same email lists. Thus, iteration was maintained by sampling from the same population but not—intentionally—by contacting the same individuals.

The same individuals were probably contacted in the two rounds and some of them probably replied in the two rounds. The IT tool used automatically recorded the internet protocol (IP) address of the respondent computer. The survey also asked a number of questions on the respondent profile such as age category. Thus, double responders were identified as those with the same profile and the same IP number. There were between 5% and 16% of double responders in the different professional categories excepting students (0.6%).

### 3.4. Correlations between Results Obtained in the Two Delphi Rounds of the Large Panel

**Figure 1:**
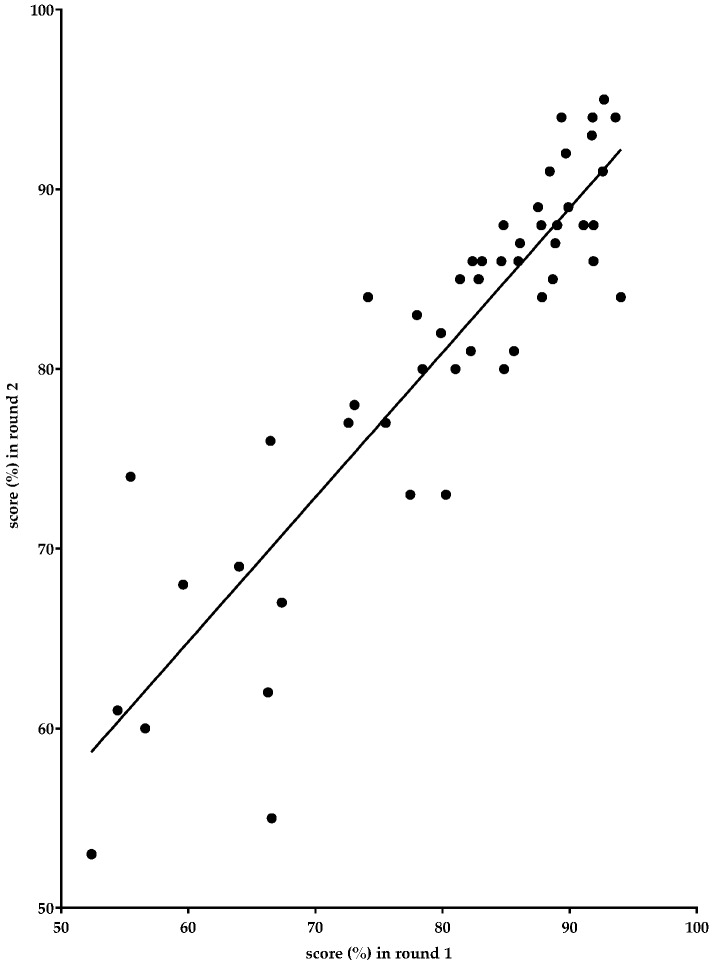
Global rankings of competences in the two rounds of the large panel Delphi process (for original see [2]).

Figure 1 below shows the global rankings for the competences in rounds one and two. The ranking is very similar in the two rounds, showing that in spite of the fact that not exactly the same populations were questioned in the two rounds, the technique used—sampling from the same listings in the two rounds—allowed the confirmation of the rankings in the second round.

The Spearman correlation between the scores in the two rounds was 0.881 (*p* < 0.0001).

### 3.5. Biases

**Figure 2:**
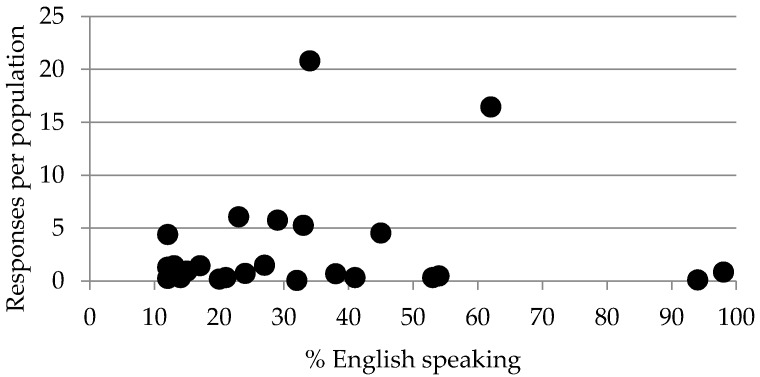
Number of respondents to the PHAR-QA (Quality Assurance in European Pharmacy Education and Training) survey in various countries versus the capacity of the population to speak English in the same country.

One possible bias may arise from the use of English which is only one of the 24 official languages in the EU. In the United Kingdom and Ireland, more than 95% of the population understands English and in some Scandinavian countries, such as Sweden and Finland, half of the population understands English. However, in southern European countries such as Spain and Portugal, less than 15% understands English [11]. No data is available as to what percentage of pharmacists understands English in various European countries. Albeit, a plot of “number of responses” versus “capacity to speak English” (Figure 2) shows no relation between the two factors. This suggests that contributions from member states with a large percentage of the population capable of understanding English were not systematically greater than those from member states with a small percentage of the population capable of understanding English. In other words, it appears that the capacity to speak English did not introduce a bias in the conclusions drawn.

Responses per population = (total number of responses from pharmacy professionals (without students)/population of the country) × 1,000,000).

% English speaking = % of people in a given country who understand English well enough to follow the news on the radio or television [11].

Spearman correlation: 0.082 (*p* > 0.05).

Several strategies were used to minimize other biases. For instance, the small panel producing the survey to be examined by the large panel, examined the formulation of questions to avoid “leading questions” i.e., suggestive interrogation evoking a particular answer from a particular subgroup.

Other biases could have arisen from the way in which respondents were approached. In an attempt to avoid bias from partial “selected” responses, we sent the questionnaire to general populations of defined representative subgroups rather than to individuals. However, this by itself could have introduced a bias. The choice of the “representative subgroup” is crucial here. For instance, we used national student associations to contact students rather than sending the questionnaire to global listings of students, the latter being not always available in all countries. Thus, we harvested results from students motivated to join a student union. The counter argument here is that such students may well be the ones interested in change and evolution in PET. Furthermore, there may well be self-selection bias by respondents themselves with selection of those more concerned with the future of pharmacy. This may be desirable if the purpose of the Delphi procedure is to direct future developments rather than to confirm present opinions.

## 4. Conclusions and Perspectives

The main element of the PHAR-QA paradigm and methodology was the use of a two-panel ranking system to both establish a highly ranked competence framework, and to ensure the transfer of the latter to the end users i.e., the professional pharmacy community. This methodology is presented here with the objective of giving readers ideas as to the ways in which to produce competence frameworks.

Several perspectives are now open. Firstly, given the rapprochement of the different branches of healthcare education—for instance the introduction of the French PACES (première année commune aux études de santé or first year of healthcare studies)—it is becoming essential to produce a common competence framework for all-embracing healthcare education and practice.

Secondly, the pharmacy academic community needs to reflect on the ways in which competence frameworks could be introduced, starting with the matching of present degree courses to the competence framework.

Thirdly, the pharmacy professional community needs to reflect on how competence training can be applied in the workplace and how the professional community can interact with the academic world. One interesting aspect of this is the development of the validation of experiential learning in pharmacy. This is important in terms of the potential validation of the work experience of pharmacy technicians wishing to pursue a degree course in pharmacy. It is also important in the validation of practical experience of pharmacy students in those parts of the world where PET does not rigidly follow the model developed in Europe.

## Abbreviations

PET	pharmacy education and training
EU	European Union

## Appendix

On behalf of the PHAR-QA (Quality Assurance in European Pharmacy Education and Training) consortium:

1. Jeffrey Atkinson, Pharmacology Department, Lorraine University, 5 rue Albert Lebrun, 54000 Nancy, and Pharmacolor Consultants Nancy, 12 rue de Versigny, 54600 Villers, France; Jeffrey.atkinson@univ-lorraine.fr.
2. Kristien De Paepe, Pharmacy Faculty, Vrije Universiteit Brussel, Laarbeeklaan 103, Brussels 1090, Belgium; kdepaepe@vub.ac.be. Kristien De Paepe replaced Bartholomew Rombaut who passed away on the 23 January 2014.
3. Antonio Sánchez Pozo, Faculty of Pharmacy, University of Granada, Campus Universitario de la Cartuja s/n, Granada 18701, Spain; sanchezpster@ugr.com.
4. Dimitrios Rekkas, School of Pharmacy, National and Kapodistrian University Athens, Panepistimiou 30, Athens 10679, Greece; rekkas@pharm.uoa.gr.
5. Daisy Volmer, Pharmacy Faculty, University of Tartu, Nooruse 1, Tartu 50411, Estonia; daisy.volmer@ut.ee.
6. Jouni Hirvonen, Pharmacy Faculty, University of Helsinki, Yliopistonkatu 4, P.O. Box 33-4, Helsinki 00014, Finland; jouni.hirvonen@helsinki.fi.
7. Borut Bozic, Faculty of Pharmacy, University of Ljubljana, Askerceva cesta 7, Ljubljana 1000, Slovenia; Borut.Bozic@ffa.uni-lj.si.
8. Agnieska Skowron, Pharmacy Faculty, Jagiellonian University, Golebia 24, Krakow 31-007, Poland; askowron@cm-uj.krakow.pl.
9. Constantin Mircioiu, Pharmacy Faculty, University of Medicine and Pharmacy “Carol Davila” Bucharest, Dionisie Lupu 37, Bucharest 020021, Romania; constantin.mircioiu@yahoo.com.
10. Annie Marcincal, Faculty of Pharmacy, European Association of Faculties of Pharmacy, Université de Lille 2, Lille 59000, France; annie.marcincal@pharma.univ-lille2.fr.
11. Andries Koster, Department of Pharmaceutical Sciences, European Association of Faculties of Pharmacy, Utrecht University, PO Box 80082, 3508 TB Utrecht, The Netherlands; A.S.Koster@uu.nl.
12. Keith Wilson, School of Life and Health Sciences, Aston University, Birmingham B47ET, UK; k.a.wilson@aston.ac.uk.
13. Chris van Schravendijk, Medical Faculty, Vrije Universiteit Brussel, Laarbeeklaan 103, 1090 Brussels, Belgium; chris.Van.Schravendijk@telenet.be.
14. Lea Noel, Pharmacy Faculty, Vrije Universiteit Brussel, Laarbeeklaan 103, 1090 Brussels, Belgium; Lea.Noel@vub.ac.be (consortial secretary).

With the assistance of:

1. Richard Price, the European Hospital Pharmacists’ Association. Available online: http://www.eahp.eu/ (accessed on13 January 2017).
2. Jamie Wilkinson, the Pharmacists’ Group of the European Union. Available online: http://www.pgeu.eu/fr/accueil.html (accessed on13 January 2017).
3. Jane Nicholson, European Industrial Pharmacists’ Group. Available online: http://eipg.eu/ (accessed on 13 January 2017).
4. The President, European Pharmacy Students’ Association, Available online: http://eafponline.eu/ (accessed on 13 January 2017).
5. The President, European Association of Faculties of Pharmacy. Available online: http://eafponline.eu/ (accessed on 13 January 2017).
